# Correction: Wu et al. Expression of MALAT1 Promotes Trastuzumab Resistance in HER2 Overexpressing Breast Cancers. *Cancers* 2020, *12*, 1918

**DOI:** 10.3390/cancers16051026

**Published:** 2024-03-01

**Authors:** Yanyuan Wu, Marianna Sarkissyan, Ochanya Ogah, Juri Kim, Jaydutt V. Vadgama

**Affiliations:** 1 Division of Cancer Research and Training, Department of Medicine, Charles R. Drew University of Medicine and Science, 1731 East 120th Street, Los Angeles, CA 90059, USA; 2 Jonsson Comprehensive Cancer Center, David Geffen School of Medicine, University of California at Los Angeles, Los Angeles, CA 90095, USA

In the original publication, there was a mistake in Figure 4B as published [1]. The authors inadvertently made an error in selecting the pAkt and T-AKT for the SKBR3/V and SKBR3/AA data in the left panel of Figure 4B during the selection of blots for publication. The corrected left panel of Figure 4B and the complete Figure 4B are shown below. 

In the original publication, there was a mistake in Figure 6B as published [1]. A human error occurred in the original Figure 6B by misplacing animal and tumor images as examples of tumor sizes between mice injected with MB231-pCDNA3 and MB231-FOXO1-4. The corrected Figure 6B, presenting the examples of tumor sizes between mice injected with MB231-pCDNA3 and MB231-FOXO1-4, and the complete Figure 6 are displayed below. 

The authors apologize for any inconvenience caused and state that the scientific conclusions are unaffected. This correction was approved by the Academic Editor. The original publication has also been updated.

## Figures and Tables

**Figure 4:**
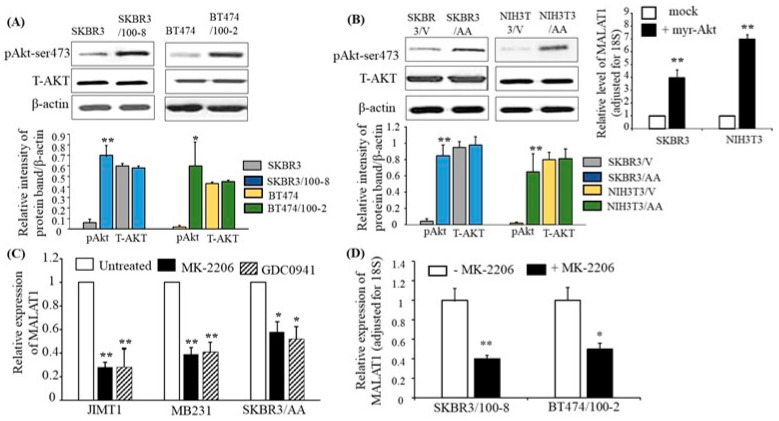
The PI3K/Akt pathway mediates the expression of MALAT1. (**A**) Total protein was extracted from each cell line, and Western blot analysis was performed with antibodies specific to phosphorylated Akt at ser-473 (pAkt) and total AKT (T-AKT). β-actin was used for loading control. The top panel shows representative Western blot images, and the bottom panel shows quantification by densitometric analysis of Western blots from four independent experiments. pAkt and T-AKT protein levels are shown relative to β-actin. The bars indicate mean ± SEM. Increased pAkt was observed in the trastuzumab-resistant cells compared to their parental lines, respectively (* *p* < 0.05 and ** *p* < 0.01). An ANOVA test was used to determine significance. (**B**) SKBR3 and NIH3T3 were stably transfected with myr-Akt (+myr-Akt) or with vector only (mock). The Western blot images in the top-left panel show the representative protein expressions of pAkt, T-AKT, and β-actin. The bottom panel shows quantification by densitometric analysis of protein (pAkt/β-actin or T-AKT/β-actin) levels from four different Western blots. The bars indicate mean ± SEM and demonstrate elevated pAkt in SKBR3/AA and NIH3T3/AA (** *p* < 0.01) compared to the respective SKBR3/V and NIH3T3/V cell lines. An ANOVA test was used to determine significance. The top-right panel shows the levels of MALAT1 relative to 18S RNA, as determined by RT-qPCR. The bar graph indicates the mean ± SEM from four independent experiments. Myr-Akt-transfected cells show increased MALAT1 expression, ** *p* < 0.01, compared to mock cells. Significance was determined using the ANOVA test. (**C**) The indicated cell lines were treated with MK-2206 or GDC0941 for 48 h, and RT-qPCR was used to determine MALAT1 relative to 18S expression. Each bar indicates mean ± SEM from four independent experiments. The data showed that MK-226 and DGC0941 inhibited expression of MALAT1 in JIMT, MB231, and SKBR3/AA cells (* *p* < 0.05 and ** *p* < 0.01) compared with their respective untreated cells. An ANOVA test was used to determine significance. (**D**) MK-2206 inhibited MALAT1 expression in trastuzumab-resistant cells. Expression of MALAT1, as determined by RT-qPCR, in SKBR3/100-8 and BT474 cells were treated with MK-226 for 48 h. The bar graph shows the level of MALAT1 adjusted for 18S. Each bar is the mean ± SEM from four independent experiments, with * *p* < 0.05, ** *p* < 0.01 compared to their respective untreated cells as determined by the ANOVA test.

**Figure 6:**
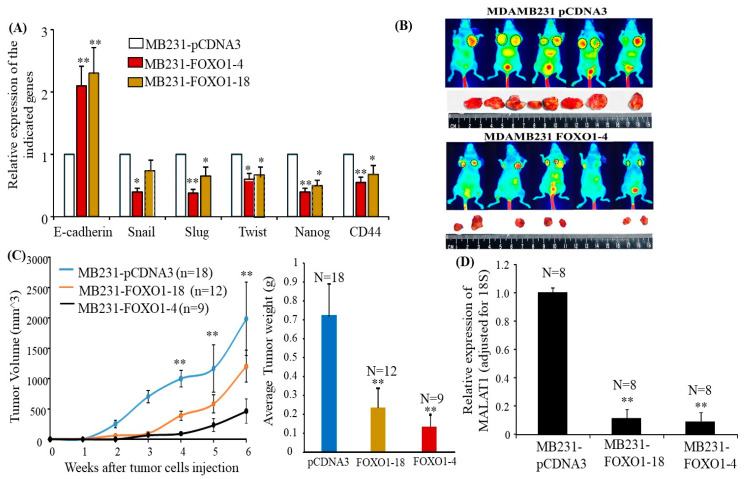
Overexpressing FOXO1 downregulates MALAT1 and inhibits tumor formation in vivo. (**A**) RT-qPCR was used to determine the expression of the indicated genes in MB231-pCDNA3, MB231-FOXO1-4, and MB231-FOXO1-18. The bar graph shows their expression levels adjusted for 18S. Each bar indicates a mean ± SEM from four repeated experiments. FOXO1 transfection reversed EMT marker gene expression, * *p* < 0.05, and ** *p* < 0.01 compared to MB231-pCDNA3 transfection as determined by ANOVA test. (**B**) The images were taken by LI-COR small-animal imaging system before tumors were removed, and the circles designate the tumor images. Representative tumors from the indicated groups are shown in (**B**). Fewer and smaller tumors were observed in the mice injected with MB231-FOXO1-4 cells. (**C**) Left panel: The indicated cells were injected into the mammary fat pads of mice as described in the Methods section. The tumor growths in mice were monitored, and tumor volumes were measured at different times. Each data point in the graph indicates mean ± SEM from the tumors detected in the respective groups. ** *p* < 0.01 compared to the tumor growth by injection with MB231-FOXO1-4 and MB231-FOXO1-18 cells, * *p* < 0.05 compared to the tumor growth by injection with MB231-FOXO1-18 and MB231-FOXO1-4 in the indicated time points. An ANOVA test was used to determine significance. Right panel: the bar graph indicates the mean ± SEM tumor weight from each group. ** *p* < 0.01 compared to the MB231-pCDNA3-cell-injected group as determined by the ANOVA test. (**D**) RNA was extracted from tumors isolated from the three groups, and RT-qPCR was used to determine MALAT1 expression in the tumor tissues. The bar graph indicates levels of MALAT1 relative to 18S. The bars indicate mean ± SEM from experiments repeated three times. MALAT1 levels were significantly lower in tumor tissues from mice injected with MB231-FOXO1-4 and MB231-FOXO1-18 cells compared to mice injected with MB231-pCDN3, ** *p* < 0.01 compared to the tumors from the MB231-pCDNA3-injected group as determined by ANOVA test.
